# *trans*-Bis(4,6-dimethyl­pyrimidine-2-thiol­ato-κ^2^ *N*,*S*)bis­(thio­urea-κ*S*)nickel(II)

**DOI:** 10.1107/S1600536809050223

**Published:** 2009-11-28

**Authors:** Jing Zhu, Jian-Gang Wang, Taike Duan, Qian-Feng Zhang

**Affiliations:** a Institute of Molecular Engineering and Applied Chemistry, Anhui University of Technology, Ma’anshan, Anhui 243002, People’s Republic of China

## Abstract

In the title complex, [Ni(C_6_H_7_N_2_S)_2_(CH_4_N_2_S)_2_], the central Ni atom (located on a centre of inversion) is six-coordinated by two monoanionic *N*,*S*-chelating 4,6-dimethyl­pyrimidine-2-thiol­ate ligands and two *trans S*-coordinating thio­urea groups. The *trans*-N_2_S_4_ donor set defines a distorted octa­hedral geometry.

## Related literature

For the significance of transition-metal complexes of heterocyclic thione ligands, see: Dilworth & Hu (1993 ▶); Figgis & Reynolds (1986 ▶); Zamudio-Rivera *et al.* (2005 ▶). For related structures, see: Rodríguez *et al.* (2007 ▶); Weininger *et al.* (1969 ▶).
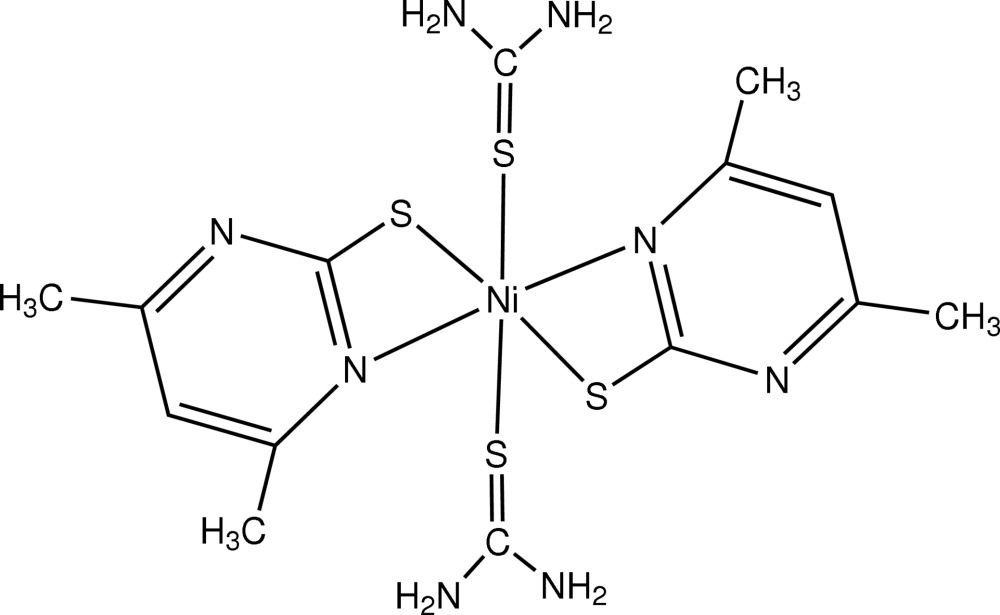


## Experimental

### 

#### Crystal data

[Ni(C_6_H_7_N_2_S)_2_(CH_4_N_2_S)_2_]
*M* *_r_* = 489.35
Orthorhombic, 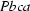

*a* = 15.0306 (2) Å
*b* = 8.5783 (1) Å
*c* = 16.9274 (2) Å
*V* = 2182.57 (5) Å^3^
*Z* = 4
Mo *K*α radiation
μ = 1.29 mm^−1^
*T* = 296 K
0.12 × 0.12 × 0.08 mm

#### Data collection

Bruker SMART CCD area-detector diffractometer
Absorption correction: multi-scan (*SADABS*; Sheldrick, 1997 ▶) *T*_min_ = 0.861, *T*_max_ = 0.901
17226 measured reflections
2495 independent reflections
1542 reflections with *I* > 2σ(*I*)
*R*_int_ = 0.061

#### Refinement

*R*[*F* ^2^ > 2σ(*F* ^2^)] = 0.040
*wR*(*F* ^2^) = 0.101
*S* = 1.07
2495 reflections
126 parameters
H-atom parameters constrained
Δρ_max_ = 0.27 e Å^−3^
Δρ_min_ = −0.30 e Å^−3^

### 

Data collection: *SMART* (Bruker, 1998 ▶); cell refinement: *SAINT-Plus* (Bruker, 1998 ▶); data reduction: *SAINT-Plus*; program(s) used to solve structure: *SHELXS97* (Sheldrick, 2008 ▶); program(s) used to refine structure: *SHELXL97* (Sheldrick, 2008 ▶); molecular graphics: *SHELXTL* (Sheldrick, 2008 ▶); software used to prepare material for publication: *SHELXTL*.

## Supplementary Material

Crystal structure: contains datablocks I, global. DOI: 10.1107/S1600536809050223/tk2572sup1.cif

Structure factors: contains datablocks I. DOI: 10.1107/S1600536809050223/tk2572Isup2.hkl

Additional supplementary materials:  crystallographic information; 3D view; checkCIF report

## Figures and Tables

**Fig. 1.**
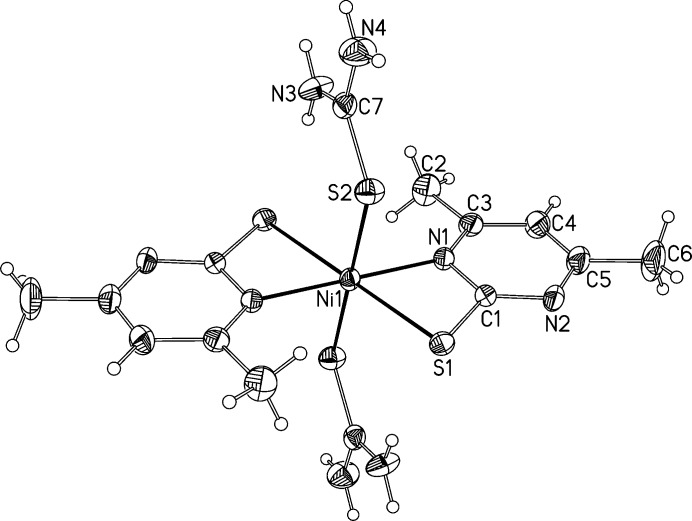
The structure of (I), showing the atom-numbering scheme and displacement ellipsoids at the 50% probability level. The Ni atom lies ona centre of inversion and unlabelled atoms are related by the symmetry operation 2-x, -y, 1-z.

## supplementary crystallographic information

### Comment

There has been extensive interest in transition metal complexes of heterocyclic thione ligands, and their thiolate derivatives, due to the potential relevance of such compounds as models of active sites in metalloenzymes and their ability to adopt structures of variable nuclearity (Dilworth & Hu, 1993). Pyrimidine-2-thione (pymtH), a typical heterocyclic thione ligand, is a versatile sulfur donor ligand in terms of coordination modes (Zamudio-Rivera *et al.*, 2005). In this paper, we report the synthesis and crystal structure of a mononuclear nickel(II) complex of 4,6-dimethylpyrimidine-2-thione (dmpymtH), namely *trans*-Ni(NH_2_CSNH_2_)_2_(dmpymt)_2_, (I).

In (I), Fig. 1, the nickel atom is located on a centre of inversion. The monoanionic dmpymt ligand functions as a chelating ligand through the S atom and one of the N atoms to form a four-membered NiSCN chelate ring. The Ni—S(dmpymt) and Ni—N bond lengths are 2.4798 (7) and 2.060 (2) Å, respectively. The N—Ni—S(dmpymt) chelate angle of 68.83 (6) ° is similar to those found in the other hexacoordinate metal complexes containing anionic heterocyclic thiolate *N*,*S*-chelate ligands (Rodríguez *et al.*, 2007). The heterocyclic thiolate ligand is essentially planar with a maximum deviation of 0.009 (2) Å from the least-squares plane for atom N1. The thiourea ligand is terminally bound to the Ni atom via coordination of the S2 atom. The Ni—S(thiourea) bond length (2.4888 (8) Å) is similar to those in *trans*-NiCl_2_(NH_2_CSNH_2_)_4_ (2.470 (1) Å) (Figgis & Reynolds, 1986) and [Ni(NH_2_CSNH_2_)_6_]Br_2_ (2.506 (1) Å) (Weininger *et al.*, 1969).

### Experimental

Treatment of a mixture of dmpymt (28 mg, 0.20 mmol) and thiourea (16 mg, 0.20 mmol) in methanol (10 ml) with Ni(NO_3_)_2_.6H_2_O (30 mg, 0.10 mmol) in methanol (10 ml) gave a light-green solution. The homogeneous solution was stirred for 2 h at 60 °, and then filtered. Slow evaporation of the solvent gave a green solid, which was recrystallized from CH_2_Cl_2_/Et_2_O to give dark-green blocks of (I) Yield: 48 mg, *ca*. 46% (based on Ni). Anal. Calcd. for C_14_H_22_N_8_NiS_4_: C, 34.4; H, 4.53; N, 22.9%. Found: C, 34.2; H, 4.50; N, 22.3%.

### Refinement

The N-bound H atoms were located in a difference map but refined with N—H = 0.86 Å, and with *U*_iso_(H) = 1.2*U*_eq_(N). The remaining H atoms were positioned geometrically and refined using a riding model, with C—H = 0.93–0.96 Å and with *U*_iso_(H) = 1.2-1.5*U*_eq_(C).

### Crystal data

**Table:**

[Ni(C_6_H_7_N_2_S)_2_(CH_4_N_2_S)_2_]	*F*(000) = 1016
*M**_r_* = 489.35	*D*_x_ = 1.489 Mg m^−^^3^
Orthorhombic, *P**b**c**a*	Mo *K*α radiation, λ = 0.71073 Å
Hall symbol: -P 2ac 2ab	Cell parameters from 2124 reflections
*a* = 15.0306 (2) Å	θ = 2.4–20.9°
*b* = 8.5783 (1) Å	µ = 1.29 mm^−^^1^
*c* = 16.9274 (2) Å	*T* = 296 K
*V* = 2182.57 (5) Å^3^	Bar, green
*Z* = 4	0.12 × 0.12 × 0.08 mm

### Data collection

**Table:**

Bruker SMART CCD area-detector diffractometer	2495 independent reflections
Radiation source: fine-focus sealed tube	1542 reflections with *I* > 2σ(*I*)
graphite	*R*_int_ = 0.061
phi and ω scans	θ_max_ = 27.5°, θ_min_ = 2.4°
Absorption correction: multi-scan (*SADABS*; Sheldrick, 1997)	*h* = −12→19
*T*_min_ = 0.861, *T*_max_ = 0.901	*k* = −11→11
17226 measured reflections	*l* = −21→21

### Refinement

**Table:**

Refinement on *F*^2^	Primary atom site location: structure-invariant direct methods
Least-squares matrix: full	Secondary atom site location: difference Fourier map
*R*[*F*^2^ > 2σ(*F*^2^)] = 0.040	Hydrogen site location: inferred from neighbouring sites
*wR*(*F*^2^) = 0.101	H-atom parameters constrained
*S* = 1.07	*w* = 1/[σ^2^(*F*_o_^2^) + (0.0412*P*)^2^] where *P* = (*F*_o_^2^ + 2*F*_c_^2^)/3
2495 reflections	(Δ/σ)_max_ = 0.001
126 parameters	Δρ_max_ = 0.27 e Å^−^^3^
0 restraints	Δρ_min_ = −0.30 e Å^−^^3^

### Special details

**Table:**

Geometry. All e.s.d.'s (except the e.s.d. in the dihedral angle between two l.s. planes) are estimated using the full covariance matrix. The cell e.s.d.'s are taken into account individually in the estimation of e.s.d.'s in distances, angles and torsion angles; correlations between e.s.d.'s in cell parameters are only used when they are defined by crystal symmetry. An approximate (isotropic) treatment of cell e.s.d.'s is used for estimating e.s.d.'s involving l.s. planes.
Refinement. Refinement of *F*^2^ against ALL reflections. The weighted *R*-factor *wR* and goodness of fit *S* are based on *F*^2^, conventional *R*-factors *R* are based on *F*, with *F* set to zero for negative *F*^2^. The threshold expression of *F*^2^ > σ(*F*^2^) is used only for calculating *R*-factors(gt) *etc*. and is not relevant to the choice of reflections for refinement. *R*-factors based on *F*^2^ are statistically about twice as large as those based on *F*, and *R*- factors based on ALL data will be even larger.

### Fractional atomic coordinates and isotropic or equivalent isotropic displacement parameters (Å^2^)

**Table:**

	*x*	*y*	*z*	*U*_iso_*/*U*_eq_	
Ni1	1.0000	0.0000	0.5000	0.03228 (16)	
S1	1.01100 (5)	0.11627 (9)	0.36624 (4)	0.0379 (2)	
S2	0.99725 (5)	0.28135 (9)	0.53580 (5)	0.0452 (2)	
N1	0.87628 (14)	0.0228 (2)	0.44894 (13)	0.0335 (5)	
N2	0.83917 (16)	0.1316 (3)	0.32332 (13)	0.0456 (6)	
N3	0.92492 (18)	0.2365 (3)	0.67697 (14)	0.0571 (8)	
H3A	0.9032	0.2694	0.7209	0.068*	
H3B	0.9267	0.1382	0.6672	0.068*	
N4	0.9512 (2)	0.4872 (3)	0.64286 (17)	0.0654 (8)	
H4A	0.9291	0.5162	0.6873	0.078*	
H4B	0.9707	0.5556	0.6100	0.078*	
C1	0.89783 (18)	0.0895 (3)	0.37873 (16)	0.0336 (6)	
C2	0.7691 (2)	−0.0829 (5)	0.54075 (19)	0.0657 (10)	
H2A	0.8090	−0.1687	0.5491	0.099*	
H2B	0.7090	−0.1207	0.5394	0.099*	
H2C	0.7754	−0.0091	0.5830	0.099*	
C3	0.7906 (2)	−0.0057 (3)	0.46408 (18)	0.0428 (8)	
C4	0.7263 (2)	0.0366 (4)	0.40970 (19)	0.0575 (10)	
H4	0.6664	0.0186	0.4200	0.069*	
C5	0.7525 (2)	0.1057 (4)	0.34018 (18)	0.0554 (9)	
C6	0.6865 (2)	0.1604 (5)	0.2792 (2)	0.0953 (15)	
H6A	0.7143	0.2356	0.2453	0.143*	
H6B	0.6364	0.2074	0.3052	0.143*	
H6C	0.6667	0.0731	0.2484	0.143*	
C7	0.9555 (2)	0.3363 (4)	0.62490 (17)	0.0416 (7)	

### Atomic displacement parameters (Å^2^)

**Table:**

	*U*^11^	*U*^22^	*U*^33^	*U*^12^	*U*^13^	*U*^23^
Ni1	0.0289 (3)	0.0400 (3)	0.0280 (3)	0.0001 (2)	0.0017 (2)	0.0070 (2)
S1	0.0374 (4)	0.0437 (4)	0.0327 (4)	−0.0003 (4)	0.0069 (3)	0.0059 (3)
S2	0.0548 (5)	0.0385 (4)	0.0423 (5)	−0.0011 (4)	0.0081 (4)	0.0016 (3)
N1	0.0282 (12)	0.0417 (14)	0.0304 (13)	−0.0005 (11)	0.0009 (10)	0.0044 (11)
N2	0.0409 (15)	0.0643 (18)	0.0317 (13)	0.0069 (13)	−0.0066 (12)	0.0038 (12)
N3	0.085 (2)	0.0480 (16)	0.0380 (15)	−0.0051 (16)	0.0154 (15)	−0.0092 (13)
N4	0.087 (2)	0.0444 (18)	0.0643 (19)	0.0023 (16)	0.0046 (18)	−0.0123 (14)
C1	0.0341 (16)	0.0353 (16)	0.0314 (15)	0.0052 (13)	−0.0019 (13)	−0.0016 (13)
C2	0.044 (2)	0.097 (3)	0.056 (2)	−0.014 (2)	0.0104 (18)	0.019 (2)
C3	0.0318 (17)	0.059 (2)	0.0375 (17)	−0.0024 (15)	0.0056 (14)	0.0005 (15)
C4	0.0306 (18)	0.092 (3)	0.050 (2)	0.0030 (18)	−0.0033 (16)	−0.0025 (19)
C5	0.040 (2)	0.084 (3)	0.0422 (18)	0.0046 (19)	−0.0097 (15)	−0.0002 (18)
C6	0.055 (2)	0.167 (4)	0.064 (2)	0.012 (3)	−0.025 (2)	0.024 (3)
C7	0.0420 (18)	0.0407 (17)	0.0421 (17)	0.0009 (15)	−0.0100 (15)	−0.0058 (16)

### Geometric parameters (Å, °)

**Table:**

Ni1—N1^i^	2.060 (2)	N4—C7	1.330 (3)
Ni1—N1	2.060 (2)	N4—H4A	0.8600
Ni1—S1	2.4798 (7)	N4—H4B	0.8600
Ni1—S1^i^	2.4798 (7)	C2—C3	1.493 (4)
Ni1—S2	2.4888 (8)	C2—H2A	0.9600
Ni1—S2^i^	2.4888 (8)	C2—H2B	0.9600
S1—C1	1.729 (3)	C2—H2C	0.9600
S2—C7	1.700 (3)	C3—C4	1.383 (4)
N1—C3	1.336 (3)	C4—C5	1.375 (4)
N1—C1	1.358 (3)	C4—H4	0.9300
N2—C1	1.337 (3)	C5—C6	1.506 (4)
N2—C5	1.352 (4)	C6—H6A	0.9600
N3—C7	1.312 (4)	C6—H6B	0.9600
N3—H3A	0.8600	C6—H6C	0.9600
N3—H3B	0.8600		
			
N1^i^—Ni1—N1	180.0	N2—C1—N1	124.8 (2)
N1^i^—Ni1—S1	111.17 (6)	N2—C1—S1	121.8 (2)
N1—Ni1—S1	68.83 (6)	N1—C1—S1	113.42 (19)
N1^i^—Ni1—S1^i^	68.83 (6)	C3—C2—H2A	109.5
N1—Ni1—S1^i^	111.17 (6)	C3—C2—H2B	109.5
S1—Ni1—S1^i^	180.0	H2A—C2—H2B	109.5
N1^i^—Ni1—S2	90.28 (6)	C3—C2—H2C	109.5
N1—Ni1—S2	89.72 (6)	H2A—C2—H2C	109.5
S1—Ni1—S2	80.41 (3)	H2B—C2—H2C	109.5
S1^i^—Ni1—S2	99.59 (3)	N1—C3—C4	119.8 (3)
N1^i^—Ni1—S2^i^	89.72 (6)	N1—C3—C2	117.2 (3)
N1—Ni1—S2^i^	90.28 (6)	C4—C3—C2	123.0 (3)
S1—Ni1—S2^i^	99.59 (3)	C5—C4—C3	118.9 (3)
S1^i^—Ni1—S2^i^	80.41 (3)	C5—C4—H4	120.6
S2—Ni1—S2^i^	180.0	C3—C4—H4	120.6
C1—S1—Ni1	76.66 (9)	N2—C5—C4	121.8 (3)
C7—S2—Ni1	119.41 (11)	N2—C5—C6	116.0 (3)
C3—N1—C1	118.3 (2)	C4—C5—C6	122.1 (3)
C3—N1—Ni1	140.6 (2)	C5—C6—H6A	109.5
C1—N1—Ni1	101.06 (16)	C5—C6—H6B	109.5
C1—N2—C5	116.3 (3)	H6A—C6—H6B	109.5
C7—N3—H3A	120.0	C5—C6—H6C	109.5
C7—N3—H3B	120.0	H6A—C6—H6C	109.5
H3A—N3—H3B	120.0	H6B—C6—H6C	109.5
C7—N4—H4A	120.0	N3—C7—N4	117.7 (3)
C7—N4—H4B	120.0	N3—C7—S2	123.0 (2)
H4A—N4—H4B	120.0	N4—C7—S2	119.3 (2)

Symmetry codes: (i) −*x*+2, −*y*, −*z*+1.
